# Organellar electrophysiology: DNA nanodevices charging at the unmeasured

**DOI:** 10.1186/s12915-023-01802-z

**Published:** 2024-01-26

**Authors:** Senthil Arumugam

**Affiliations:** 1 Monash Biomedicine Discovery Institute, Faculty of Medicine, Nursing and Health Sciences, Monash University, Clayton, Melbourne, VIC 3800 Australia; 2 European Molecular Biology Laboratory Australia (EMBL Australia), Monash University, Clayton, Melbourne, VIC 3800 Australia

## Abstract

**Organellar ion channels are regulators of key processes of cellular homeostasis as well as being involved in diseases including cancer, neurological disorders and virus infection. Individual organelle-level electrophysiology has until recently only been performed using isolated organelles, removing any direct physiological context. New DNA-based nanodevices — Clensor (chloride sensor), RatiNa (sodium sensor) and pHlicKer (potassium sensor), now allow imaging-based mapping of sodium and potassium levels in membranous organelles. This breakthrough paves the way for studying ion homeostasis relevant to a variety of diseases as well as fundamental processes.**

## Why do ions matter at organelles?

Ions are crucial for cell physiology, with one aspect of it being gradients across organellar membranes that govern the function of various proteins as well as overall transport characteristics for specific cargoes. Malfunctioning of ion channels results in diseases such as lysosomal storage disorders, metabolic pathologies and neurodegenerative diseases. Over the last decades, genes of several intracellular ion channels have been identified. Electrophysiological characterisation of organelle-localised ion channels through classical patch clamping on extracted organelles has allowed characterising their activity. A few studies strongly suggest a direct physiological role of ion channels at the endosomal-lysosomal (endolysosomal) pathways. In studies linking phosphatidylinositol 3,5-bisphosphate (PI(3,5)P_2_) and Na^+^ channel, TPC2 (two-pore channel 2) activation suggests that the level of specific phosphoinositides [1], a mark of endosomal maturation, may also functionally link to ion homeostasis.

Multiple ion channels and transporters are shown to be involved in processes regulated at the endosomal network including virus trafficking. *Bunyamwera* virus traffics through endosomes with high K^+^, which directly affects the infectivity of the virions in A549 cells. K^+^ channel inhibition alters the distribution of bunyaviruses and arrests their trafficking [2]. Multiple ion channels and transporters control luminal ionic concentrations at the endosomes. Mutations in TRPML1, also activated by PI(3,5)P_2_, result in conditions of lysosomal storage disorder such as mucolipidosis-type 4.

In immune cells, nascent macropinosomes fuse with various endosomes, which determine subsequent pathways of degradation or recycling routes. One of the features of macropinosomes post fusion is that they decrease in volume as well as undergo fragmentation, termed ‘macropinosome resolution’. This process requires efflux of ions as well as osmotic balance with water. Osmotic changes due to misregulation of ion concentrations may also affect morphological distortions such as tubulation or invagination, which in turn can affect cargo itinerary. Generally, ionic strength can also affect bilayer structure and curvature, resulting in preference to a set of proteins that have curvature recognition domains. A more detailed review detailing ion flux and the function of endosomes and lysosomes can be found here [3].

## A new paradigm of organellar electrophysiology

Subcellular compartments — or *organelles* — were first described in 1950s. Since then, there has been much interest in understanding intracellular trafficking between organelles and cell membrane. Key discoveries included lysosomes, sec genes that encode secretory proteins, the SNARE complex mediating vesicle fusion, and the role of calcium ions and calcium-sensitive proteins in synaptic fusion. The process of luminal acidification was more prominently featured with the discovery of V-type proton ATPase. While proton and calcium gained prominence, only in the last few decades, several other endolysosomal ion channels have been characterised. This was enabled by the patch-clamp technique, a refined electrophysiological method developed by Sakmann and Neher in 1984 for characterising ion channels on the plasma membrane of the cell [4]. Organelle electrophysiology is difficult to achieve, as the size of many membranous organelles is less than a micron in diameter and organelles must be isolated in some manner while maintaining their integrity. Despite these challenges, patch clamping has been applied to organelles to characterise ion channels [5]. However, the biggest drawback of the method thus far is that the quintessential factors affecting organelle biology — rapid dynamics such as movement, interaction with other organelles, fusion, fission and ever-changing biochemical environment — are all lost when organelles are isolated. This is where imaging-based ion measurements, which can measure organellar ion contents in their native, dynamic environment — have been transformative.

The imaging-based approach for sensing ion concentration opens the possibility of studying the function of ion channels in organelle-organelle interactions, as well as the spatiotemporal dynamics within organelles and in conjunction with other localised biochemical reactions. Recently developed DNA-based nanodevices have enabled researchers to perform ‘electrophysiology’ of discrete membranous compartments in their native physiological environments using microscopy. DNA-based nano-devices are designed by integrating multiple modules that perform distinct functions — the chief modules being targeting and sensing. These DNA-based nanodevice sensors include chloride, sodium and potassium sensors; Clensor was demonstrated to detect chloride levels in lysosomes [6], while in recent publications, two novel sensors RatiNa (sodium sensor) [7] and pHlicKer (potassium sensor) [8] were described. These sensors can perform measurements independent of the pH and report of sodium [7] or potassium [8] ion concentrations in live, dynamic endolysosomes. This offers a unique opportunity to perform ‘electrophysiology’ of discrete membranous compartments in their native physiological environments using microscopy, a significant breakthrough from previous state of the art of isolating organelles and performing laborious patch-clamp electrophysiology.

## Imaging approaches for multiplexed measurements

The endolysosomal compartments are a dynamic set of compartments that undergo a multitude of dynamic events. Morphologically, they undergo fusion, fission, tubulation and invaginations leading into multi-vesicular bodies. Biochemically, their lipidic composition alters, chiefly via conversion of phosphoinositides that attract specific proteins with specific binding domains or via tubulation and scission, where distinct lipid populations may be sorted. A common approach of defining these populations is to identify the specific proteins that are found on them, the chief ones belonging to the Rab family of proteins. Given the complexity and the stochastic nature of events (events that seemingly appear by chance) in the endolysosomal system, measuring just ionic concentrations is not sufficient to provide context of their role in a specific process, and it is imperative that multiple measurements — e.g. for endosomal markers or cargoes — are taken in parallel to correlate events. Addressing this issue, recent advances in light-sheet microscopy with capability of rapid, volumetric imaging with subcellular resolution, complemented with computational image analysis, can enable capturing rich sets of data, despite the stochastic nature of endosomal events [9]. Furthermore, imaging modalities like multispectral and hyperspectral approaches [10] will enable simultaneous measurements of multiple lipid or proteins species along with ion concentrations, revealing new details about the dynamics of ion fluxes.

## Conclusions

Given the central role of ions as exemplified with a few mentions above, the advent of technical capabilities in imaging and analysis, along with the recently described DNA-nanodevice sensors, now opens the field for exploring the unknown on ionic fluxes in the endolysosomal system. Future versions that may offer prolonged high-temporal resolution imaging may reveal unknown features of ionic changes in the endolysosomal system. While these changes may not be as rapid as the action potentials, timely ionic fluxes in the endolysosomal system may play an important role in regulation of cell homeostasis, given that aberrant functioning of many of these channels results in human diseases. Thus, these new generation sensors open a new door to explore physiology, disease pathophysiology and pharmacology of more than 70 different ion channels that are harboured in the endolysosomal system.

## Data Availability

Not applicable.
